# A Comprehensive Bioinformatic Analysis of SLC52A3 as a Prognostic Biomarker and Potential Therapeutic Target in Gynecological Cancers

**DOI:** 10.3390/genes17060669

**Published:** 2026-06-07

**Authors:** Monia Cecati, Valentina Schiavoni, Roberto Campagna, Giovanni Tossetta

**Affiliations:** 1 Department for the Promotion of Human Science and Quality of Life, San Raffaele Roma University, 00166 Rome, Italy; monia.cecati@uniroma5.it; 2 Department of Clinical Sciences, Polytechnic University of Marche, 60126 Ancona, Italy; v.schiavoni@pm.univpm.it

**Keywords:** SLC52A3, riboflavin transporter, gynecological cancers, endometrial cancer, ovarian cancer, cervical cancer, prognostic biomarker, bioinformatics, TCGA, tumor microenvironment, immune infiltration, therapeutic target

## Abstract

Background/Objectives: The gene solute carrier family 52 member 3 (SLC52A3) encodes riboflavin transporter-3, a transmembrane protein essential for riboflavin absorption. Emerging evidence suggests that metabolic transporters may play a role in tumor biology. This study aimed to investigate the expression patterns, prognostic significance, genetic alterations, and functional associations of SLC52A3 in gynecological cancers. Methods: A comprehensive bioinformatic analysis was conducted using multi-omics datasets from The Cancer Genome Atlas (TCGA). Gene expression and survival analyses were performed via GEPIA3. Genetic alterations, including mutations and copy number variations, were assessed using cBioPortal. Immune infiltration correlations were analyzed through TIMER3. Protein–protein interactions and gene enrichment analyses were performed using STRING and GEPIA2, followed by Gene Ontology (GO) and KEGG pathway analyses. Results: SLC52A3 expression was significantly upregulated in ovarian, cervical, and endometrial cancers. Reduced expression of SLC52A3 was associated with poorer overall survival and shorter progression-free interval specifically in endometrial cancer. Genetic alterations in SLC52A3 were not significantly associated with survival outcomes (OS, DFS, and PFS). Functional enrichment analysis indicated that SLC52A3 is involved in biological processes such as cell junction organization and protein localization to the plasma membrane. Additionally, SLC52A3 expression showed positive correlations with genes implicated in tumor progression and metastasis, including NECTIN4, PROM2, TACSTD2, PKP3, SEMA4B, and CD46. Conclusions: These findings suggest that SLC52A3 may serve as a potential prognostic biomarker in endometrial cancer and could play a role in tumor progression pathways. Its functional associations highlight its potential relevance as a therapeutic target, warranting further experimental validation.

## 1. Introduction

Over the past decade, multi-omics cancer datasets such as The Cancer Genome Atlas (TCGA) have been widely used in cancer research, enabling the understanding of the molecular mechanisms underlying many neoplasms [1]. Bioinformatic studies play a key role in cancer research since they allow to researchers to examine gene functions, mutations, expression profiles and survival outcomes in several malignancies. Consequently, these studies are important to find new genes that can be used as therapeutic targets in cancer treatment in order to improve the outcome of several malignancies.

Gynecological cancers include six types of cancers: cervical, ovarian, uterine (endometrial), vulvar, vaginal and fallopian tube (very rare) cancer [2]. Gynecological cancers are commonly treated with surgery, chemotherapy, radiation and targeted/immune therapies, but therapy resistance occurrence is very frequent, significantly increasing the mortality rate on these malignancies [2,3,4,5].

In this study, we performed a bioinformatic analysis on SLC52A3 in gynecological cancers using multiple databases in order to elucidate its function in these malignancies.

Gene solute carrier family 52 member 3 (SLC52A3, also known as C20orf54) encodes riboflavin transporter-3, a transmembrane protein with a key role in the absorption of riboflavin and regulation of riboflavin tissue distribution [6]. Humans, but also other mammals, cannot synthesize riboflavin endogenously and obtain riboflavin from diet and normal microflora of the large intestine [6]. It has been found that SLC52A3 is the most efficient riboflavin transporter [7], and its expression can be increased by riboflavin deficiency, suggesting an important role of this transporter in maintaining riboflavin homeostasis [8].

Several studies showed that SLC52A3 expression is significantly increased in many cancers, including cervical [9], pancreatic [10] and esophageal cancer [11] and glioma [12]. It has also been demonstrated that SLC52A3 significantly enhanced the proliferation of esophageal cancer and glioma cells, suggesting an important role of SLC52A3 in cancer progression and tumorigenesis [12,13].

The aim of this study was to conduct a systematic examination of SLC52A3 in gynecological cancers present in TCGA dataset by using bioinformatic tools in order to evaluate its expression patterns, prognostic implications, genetic mutations, and molecular roles and its interplay with the tumor microenvironment (TME) in these malignancies.

## 2. Materials and Methods

The SLC52A3 mRNA expression profile was investigated by using the Human Protein Atlas (HPA) database (version 25.0) (https://www.proteinatlas.org/). The expression differences of SLC52A3 in cancerous and non-cancerous tissues were investigated by the Tumor Immune Estimation Resource version 2 (TIMER2) (http://timer.cistrome.org/).

### 2.1. Survival Prognosis Analysis

The overall survival (OS) and disease-free survival (DFS) Kaplan–Meier (KM) plots, as well as the survival significance maps of SLC52A3 in gynecological cancers, were generated by using the “Survival Analysis” module of Gene Expression Profiling Interactive Analysis version 3 (GEPIA3) (https://gepia3.bioinfoliu.com). SLC52A3 expression across gynecological cancer stages was visualized as violin plots and created using the GEPIA3 tool [14].

### 2.2. Genetic Alteration Analysis

The cBioPortal tool (http://www.cbioportal.org; version 6.0) was used to analyze SLC52A3 genetic alterations in gynecological cancers of TCGA pan-cancer atlas. In particular, we extracted the frequency of SLC52A3 gene mutation site map using the “Mutations” module of the cBioPortal tool. In addition, we collected information about SLC52A3 mutation frequency, types, site-specific mutations and copy number variations (CNAs) [15]. Survival outcomes such as overall survival (OS), progression-free survival (PFS), and disease-free survival (DFS) were also collected.

### 2.3. Immune Cell Infiltration Analysis

The “Immune” module of the Tumor Immune Estimation Resource version 3 (TIMER3) tool “https://compbio.cn/timer3 (accessed on 4 May 2026)” was used to evaluate the correlation between SLC52A3 expression and immune infiltration [16]. The following algorithms were used to quantify the immune infiltration: TIDE, EPIC, XCELL, MCPCOUNTER and CONSENSUS_TME. To illustrate the correlation between SLC52A3 levels and immune cell types, heatmaps and scatter plots were generated.

### 2.4. SLC52A3-Related Gene Enrichment Analysis

We used the STRING database (https://cn.string-db.org; version 12.0) to identify proteins interacting with SLC52A3 [17]. A minimum interaction score of “medium confidence (0.400)” and network edges based on “evidence” from experimentally validated interactions were set. The GEPIA3 “Expression network” module was used to identify the top 100 genes correlated with SLC52A3 from the TCGA dataset. We conducted a Pearson correlation analysis between SLC52A3 and these genes, generating a dot plot of the mean log2 (TPM + 1) values. Moreover, Spearman’s rank correlation test was used for specific statistical tests Graphical visualization of the Kyoto Encyclopedia of Genes and Genomes (KEGG) and Gene Ontology (GO) enrichment analyses was generated using the SRplot online platform [18]. Bar plots were used to display significantly enriched GO terms, while the enrichment scores were expressed as –log10 (*p*-value).

### 2.5. EGFR-Protein Interaction Analysis

We used the “Network” module of BioGRID repository (version 5.0.254) (https://thebiogrid.org) to create an SLC52A3 protein interaction network.

## 3. Results

### 3.1. SLC52A3 Expression in Normal Tissues and Gynecological Cancers

**Figure 1:**
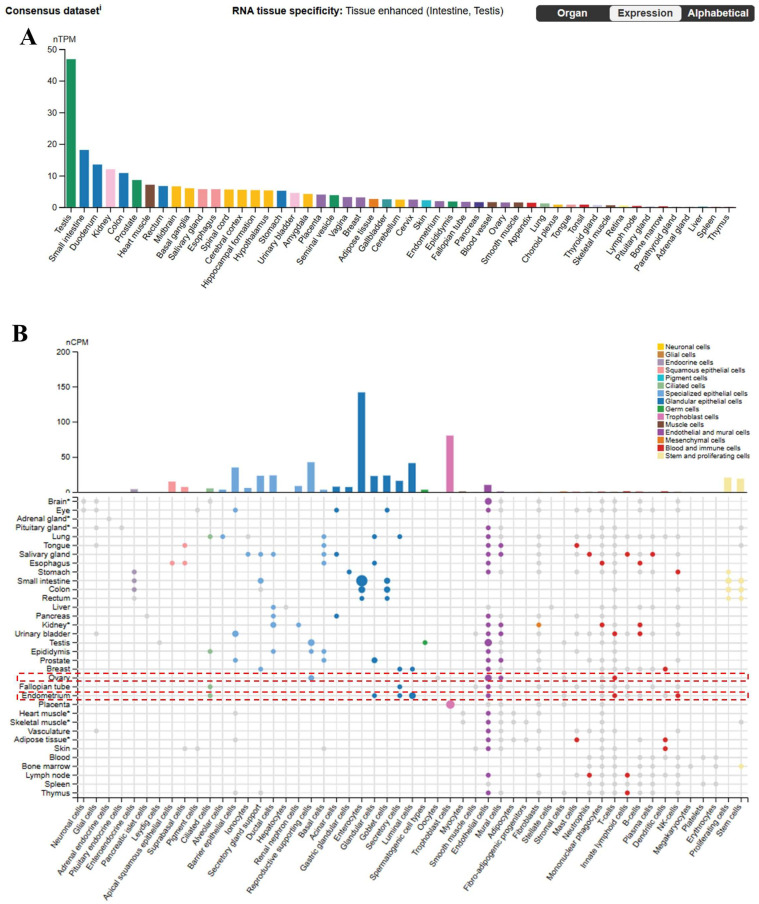
Expression status of SLC52A3 in normal tissues (**A**) and cells (**B**). The consensus dataset (**A**) consists of normalized expression (mTPM) levels of 55 tissue types created by combining the HPA and GTEx transcriptomics datasets using the internal normalization pipeline. RNA single cell type group specificity (**B**).

Using HPA and GTEx datasets, we observed a high expression of SLC52A3 in the testis, small intestine, duodenum, kidney and colon (Figure 1A). Moreover, we observed a high expression of SLC52A3 in enterocytes and trophoblast cells through single-cell RNA-seq datasets (Figure 1B). Looking at these findings, we can highlight a low tissue specificity for SLC52A3 expression.

**Figure 2:**
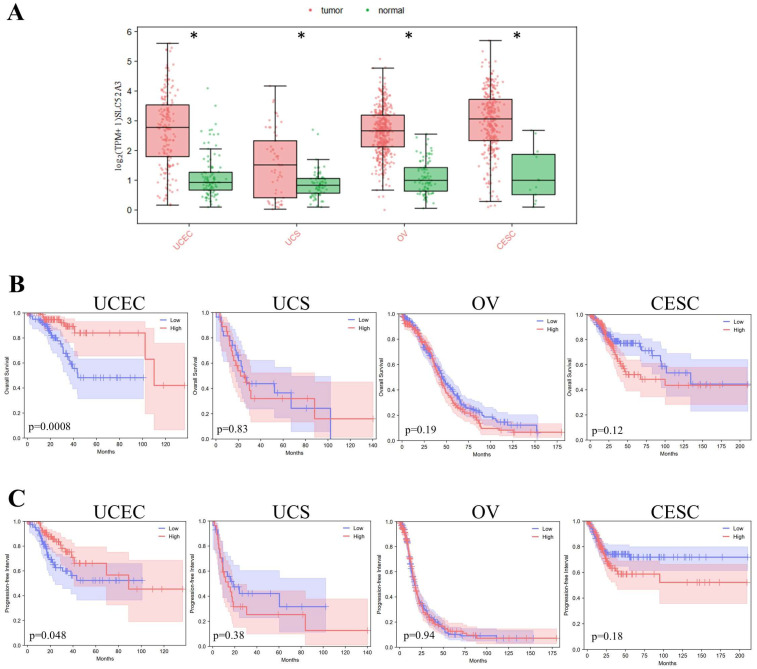
Expression status of SLC52A3 in gynecological cancers. SLC52A3 expression levels in uterine corpus endometrial carcinoma (UCEC), uterine carcinosarcoma (UCS), ovarian cancer (OV) and cervical squamous cell carcinoma (CESC) compared to their respective normal counterparts (**A**). Correlation between SLC52A3 expression and overall survival (**B**) and progression-free interval (PFI) (**C**) in UCEC, UCS, OV and CESC. * *p* < 0.05.

Subsequently, we scrutinized SLC52A3 expression patterns in gynecological cancers in order to evaluate SLC52A3 mRNA expression level variations compared to normal tissues. As shown in Figure 2A, SLC52A3 mRNA expression levels were significantly upregulated in uterine corpus endometrial carcinoma (UCEC), uterine carcinosarcoma (UCS), ovarian cancer (OV) and cervical squamous cell carcinoma (CESC) compared to their respective normal counterparts.

These findings highlight a complex role of SLC52A3 in tumorigenesis, suggesting that the expression patterns of SLC52A3 may significantly change across different cancer types and tumor stages, highlighting SLC52A3 as a potential therapeutic and prognostic target in cancer management.

### 3.2. Prognostic Role of SLC52A3 in Gynecological Cancers

To evaluate a potential prognostic role of SLC52A3 expression in gynecological cancers of the TCGA dataset, we used GEPIA3, an interactive web server, for analyzing the RNA sequencing expression data of normal and cancer tissues. As shown in Figure 2, the overall survival analysis showed the following *p*-values: *p* = 0.0008 (in UCEC), *p* = 0.83 (in UCS), *p* = 0.19 (in OV) and *p* = 0.12 (in CESC) (Figure 2B). 

In addition, progression-free interval (PFI) analysis showed that low SLC52A3 expression was associated with an adverse prognosis in patients with UCEC (*p* = 0.048), while there was no statistically significant association regarding SLC52A3 expression in CESC, OV and UCS (Figure 2C).

Thus, in UCEC, a low SLC52A3 expression is associated with a poor overall and progression-free interval. These results highlight an important role of SLC52A3 in cancer progression and patient survival, suggesting that SLC52A3 expression levels could be a useful prognostic marker in UCEC, guiding therapeutic strategies and personalized treatment approaches to improve the outcome of these patients.

**Figure 3:**
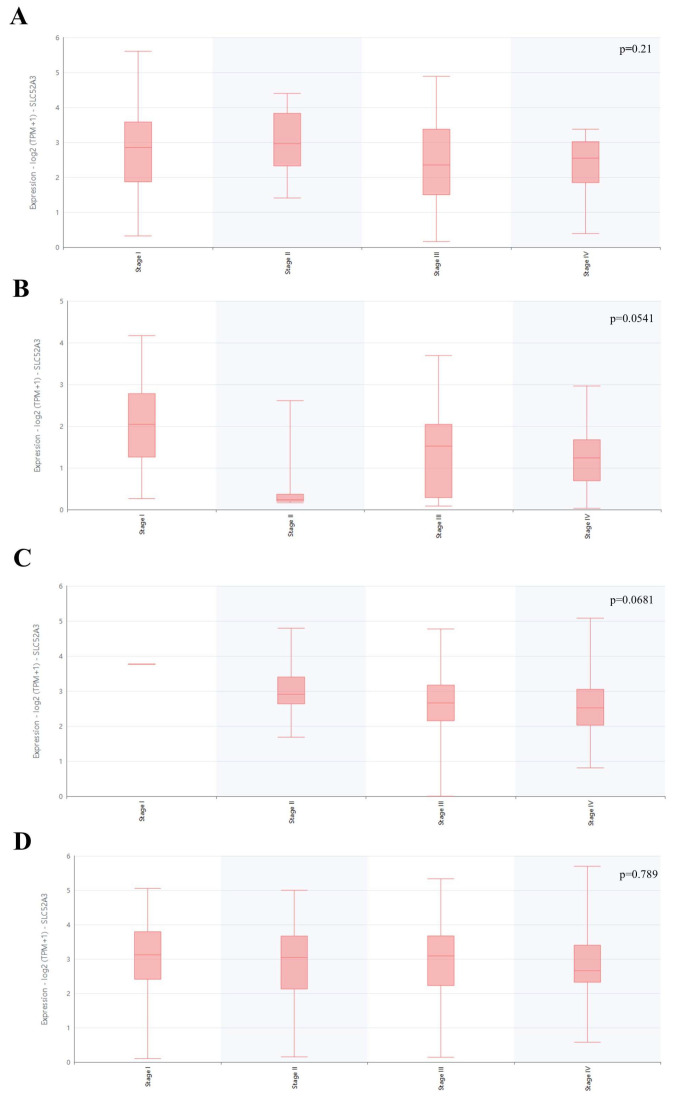
Pathological stage plot focused on SLC52A3 expression revealed no statistically significant correlation with pathological staging in UCEC (**A**), UCS (**B**), OV (**C**) and CESC (**D**). SLC52A3 expression levels were quantified using a log2 (TPM + 1) scale to normalize gene expression data and facilitate comparisons across samples.

Furthermore, we evaluated a possible correlation between SLC52A3 expression levels and advanced pathological stages in gynecological cancers of the TCGA dataset in order evaluate a possible role of SLC52A3 in tumor aggressiveness and disease progression in these malignancies. As shown in Figure 3, no statistically significant correlation with pathological staging was found in UCEC (Figure 3A), UCS (Figure 3B), OV (Figure 3C) and CESC (Figure 3D). Thus, SLC52A3 expression is not associated with tumor aggressiveness.

### 3.3. Evaluation of Genetic Alterations of SLC52A3 in Gynecological Cancers

The characterization and identification of genetic changes in specific genes play a key role in the development of targeted therapies. Thus, understanding the specific mutational patterns and their clinical implications can allow personalized treatment approaches useful for improving patient outcomes.

**Figure 4:**
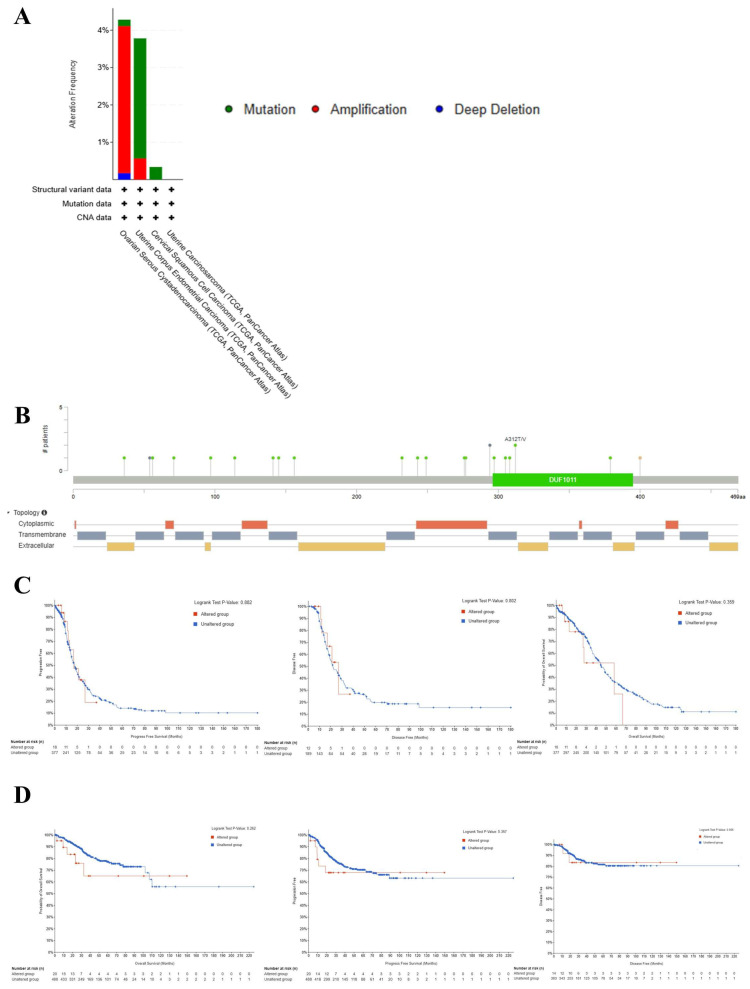
SLC52A3 gene alterations in gynecological cancers from the TCGA dataset generated using cBioPortal. Distribution of different types of genetic alterations in the SLC52A3 gene (**A**). Pinpoints of the specific mutation sites within the SLC52A3 gene (**B**). Correlation between SLC52A3 alteration status with overall survival (**B**), progression-free survival (PFS) and disease-free survival (DFS) in OV (**C**) and UCEC (**D**).

In order to evaluate genetic alterations associated with the SLC52A3 gene in gynecological cancers, we used cBioPortal for cancer genomics, an interactive platform useful for visualizing and analyzing genetic data from cancer studies. Our analysis showed that the frequency of SLC52A3 alterations in gynecological cancers is generally very low (0–4.28%) (Figure 4A). In particular, ovarian cancer samples are the most altered since the SLC52A3 gene is mutated (0.17%), amplified (3.94%) and deleted (0.17%) in these patients. Patients with endometrial carcinoma showed mutation (3.21%) and amplification (0.57%) of the SLC52A3 gene. Patients with cervical cancer showed SLC52A3 gene mutation (0.34%), while no gene alterations were found in uterine carcinosarcoma (Figure 4A).

After further examination, we found a total of 21 mutations in the SLC52A3 gene across the gynecological cancer samples. In particular, we identified the following types of mutations: 18 missense, 2 truncating and 1 splicing (Figure 4B). No inframe or fusion mutations were identified.

Since the presence of these mutations can impair function of the SLC52A3 protein, we examined the clinical implications of SLC52A3 alterations with important survival metrics such as progression-free survival (PFS), disease-free survival (DFS) and overall survival (OS) in each gynecological cancer. As shown in Figure 4, SLC52A3 alterations were not statistically significantly associated with a poor PFS, DFS or OS in OV (Figure 4C) or UCEC (Figure 4D). We were not able to perform this analysis in CESC since the SLC52A3 gene was found to be altered only in one patient, which was not enough to carry out a statistical analysis.

These findings demonstrated that SLC52A3 gene alterations are not clinically relevant and cannot be used as potential prognostic biomarkers in gynecological cancers.

### 3.4. Evaluation of Cancer-Associated Fibroblast Infiltration

**Figure 5:**
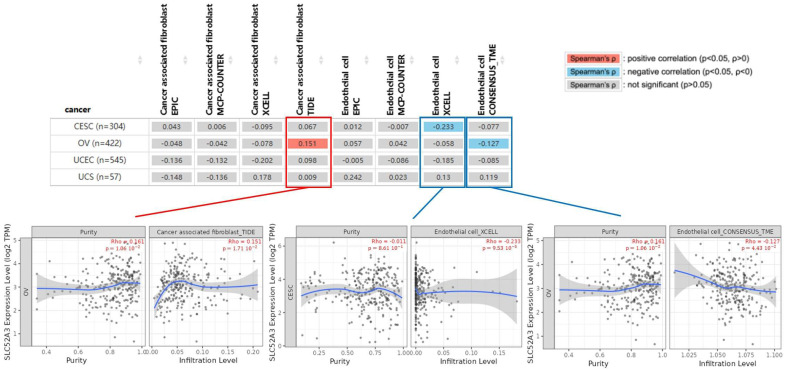
Correlation between SLC52A3 expression and the immune infiltration of cancer-associated fibroblasts (CAFs) across gynecological cancers (CESC, OV, UCEC and UCS) within the TCGA dataset using various algorithms. A positive correlation between SLC52A3 expression and CAFs was noted in OV, while a significant negative correlation was observed between SLC52A3 expression and endothelial cells in CESC and OV.

Since cancer-associated fibroblasts (CAFs) and endothelial cells play a key role in modulating the immune response within the tumor stroma and then influencing tumor progression and development, we used multiple TIMER3-based computational algorithms, including EPIC (Estimation of Per-Immune Cell), TIDE (Tumor Immune Estimation Resource), XCEL, MCP-COUNTER and the CONSENSUS_TME of TIMER3.0 webserver to evaluate the relationship between SLC52A3 expression and immune cell infiltration in gynecological cancers from the TCGA dataset. These algorithms were selected because they use different immune/stromal reference signatures and computational frameworks, allowing complementary estimation of tumor-infiltrating immune and stromal cell populations from bulk RNA-seq data. As shown in Figure 5, a positive correlation between SLC52A3 expression and CAF presence was found only in ovarian cancer patients by the TIDE algorithm. Contrarily, a negative correlation was found between SLC52A3 expression and endothelial cell presence in ovarian (OV) and cervical (CESC) cancer by CONSENSUS_TME and XCELL, respectively.

Our findings suggest a possible association between SLC52A3 expression and selected stromal components of the tumor microenvironment across gynecological cancers. Although the use of multiple algorithms provides a broader assessment of immune infiltration and reduces dependence on a single deconvolution method, correlations detected by only one algorithm should be interpreted cautiously and considered hypothesis-generating and do not demonstrate a direct functional effect of SLC52A3 on immune response or immunotherapy efficacy.

### 3.5. Enrichment Analysis of SLC52A3-Related Genes

In order to identify the top 100 genes whose expression patterns are analogous to SLC52A3 across gynecological cancers in the TCGA dataset, we used the GEPIA3 webserver to identify possible functional mechanisms by which SLC52A3 may contribute to carcinogenesis processes.

**Figure 6:**
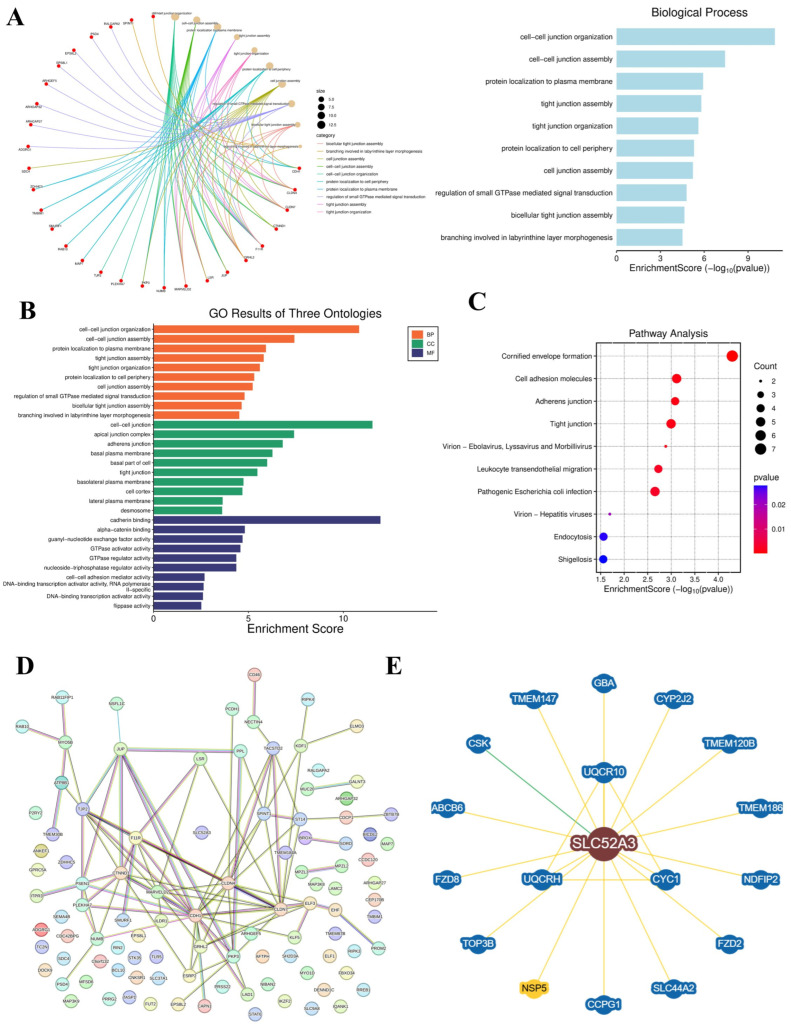
SLC52A3-related gene enrichment analysis. Correlation network of 29 genes co-expressed with SLC52A3 and enrichment degree of SLC52A3 in biological process ontology (**A**). Gene Ontology enrichment of SLC52A3 in biological process, molecular function, and cellular components (**B**). KEGG pathway analysis of top 100 genes co-expressed with SLC52A3 obtained by GEPIA3 (**C**). Co-expression network of 100 genes co-expressed with SLC52A3 obtained through the STRING tool (**D**). SLC52A3 protein interactions obtained from BioGRID (**E**).

Gene Ontology (GO) and KEGG pathway enrichment analyses were used to uncover the functional roles of SLC52A3 in cancer biology. As shown in Figure 6A,B, GO enrichment analysis of these genes showed a moderate (PCC from 0.43 to 0.47) positive association with biological processes such as cell junction organization/assembly and protein localization to the plasma membrane, indicating their involvement in SLC52A3-driven carcinogenesis. However, although the analysis revealed a good correlation among these genes (as depicted in Figure 6A,E), the STRING tool did not highlight a direct interaction of these genes with SLC52A3 (Figure 6D).

Additionally, KEGG analysis highlighted terms such as “Endocytosis”, “Tight junction” and “Adherens junction”, which could be involved in mediating SLC52A3 oncogenic actions (Figure 6C). These findings improve our understanding regarding the role of SLC52A3 in tumorigenesis and its wide influence on several cellular processes.

Data from the BioGRID (Figure 6E) database showed that SLC52A3 physically interacts with proteins such as Ubiquinol-Cytochrome C Reductase (UQCR), Complex III Subunit X (10) (UQCR10), UQCR hinge protein (H) (UQCRH) and cytochrome c-1 (CYC1), all components of Ubiquinol-cytochrome c oxidoreductase (also known as Complex III) [19]. Complex III is a key enzyme located in the inner mitochondrial membrane that plays a key role as a component of the electron transport chain since it oxidizes ubiquinol and reduces cytochrome c while pumping protons to generate an electrochemical gradient.

**Figure 7:**
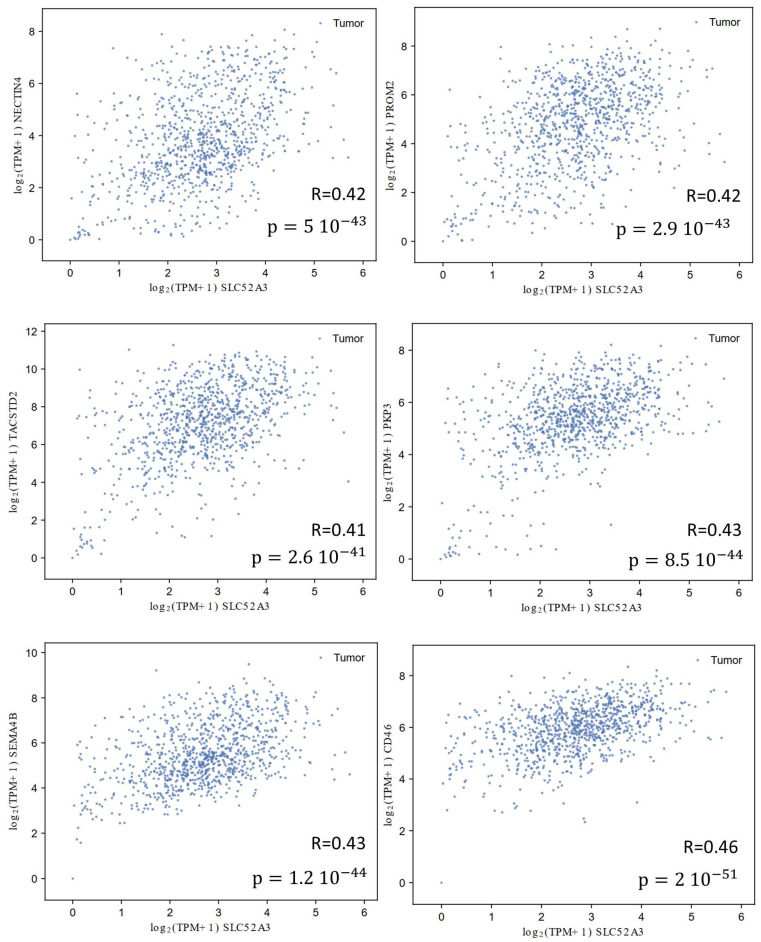
Correlation analysis between SLC52A3 and NECTIN4, PROM2, TACSTD2, PKP3, SEMA4B and CD46 across gynecological cancer samples in TCGA performed by GEPIA3.

As shown in Figure 7, among the top 100 genes, SLC52A3 expression was found to be positively correlated to the expression of several other genes involved in cancer progression, proliferation and metastasis, including NECTIN 4, PROM2, TACSTD2, PKP3, SEMA4B and CD46.

These associations further support the hypothesis that SLC52A3 may facilitate cancer cell growth.

## 4. Discussion

In this study we provided a comprehensive investigation into the role of SLC52A3 in gynecological cancers, analyzing its expression patterns in cancerous and normal tissues, genetic alterations, prognostic value and mechanistic pathways. The findings showed an important role of SLC52A3 in tumorigenesis, suggesting SLC52A3 as a potential therapeutic target in gynecological cancers.

A recent review by Ubaid et al. [20] highlighted that oncogenesis arises from coordinated alterations across multiple molecular layers such as somatic mutations, copy number changes, gene expression dysregulation, proteomic changes, and epigenetic remodeling. Moreover, they highlighted the importance of multi-omics integration as a more complete framework for identifying cancer driver genes, understanding pathway-level vulnerabilities, and refining precision oncology strategies [20]. This perspective reinforces the rationale of our study, which used bioinformatic analysis to capture oncogenic determinants that can be further studied and validated with ad hoc studies to demonstrate their potential role as therapeutic targets in cancer treatment.

Our analysis of SLC52A3 expression across gynecological cancers using TCGA_GTEx data showed a significant upregulation of SLC52A3 in UCEC, UCS, OV and CESC. The results obtained were consistent with recent studies reporting an increased expression of SLC52A3 in pancreatic cancer [10], esophageal cancer [11], cervical cancer [9] and glioma [12], suggesting that SLC52A3 may contribute to tumorigenesis. The clinical significance of SLC52A3 was further underscored by our survival analysis reporting a poor OS and PFI in UCEC patients with low SLC52A3 expression. No clinical significance was found in UCS, OV and CESC.

The poor OS and PFI in UCEC patients with low SLC52A3 expression was quite surprising since SLC52A3 expression was significantly higher in tumor tissues compared with their normal counterparts. However, this apparent paradox may be due to the metabolic role of SLC52A3 as a riboflavin transporter involved in several important cellular processes such as redox balance, flavin cofactor availability (e.g., Flavin Mononucleotide (FMN) and Flavin Adenine Dinucleotide (FAD)), and mitochondrial oxidative metabolism (ATP generation) [21,22]. It deserves to be pointed out that this observation may reflect tumor-context-dependent biology, molecular subtype heterogeneity, dedifferentiation, stromal/immune composition, or metabolic adaptation and that these possibilities cannot be resolved using bulk transcriptomic data alone. In fact, since the present analysis is based on bulk transcriptomic and correlation-based datasets, these mechanisms remain speculative and require direct functional validation using in vivo or in vitro models.

Moreover, survival analyses performed using GEPIA3 were based on Kaplan–Meier and Cox regression approaches stratified by SLC52A3 expression levels. Ad hoc studies are necessary to evaluate patient survival based on SLC52A3 expression levels adjusted for clinicopathological covariates such as age and tumor stage, grade and molecular subtypes. Therefore, the observed association between SLC52A3 expression and survival should be interpreted as exploratory and hypothesis-generating.

The increased SLC52A3 expression in cancer cells may be an adaptive response associated with carcinogenesis, and tumors with a low SLC52A3 expression may evolve toward more aggressive metabolic states. Thus, tumors with reduced SLC52A3 expression may represent more metabolically dysregulated phenotypes associated with increased aggressiveness and worse clinical outcomes. Thus, SLC52A3 could act as both a marker of metabolic adaptation and a potential indicator of tumor aggressiveness in UCEC patients. Although SLC52A3 expression could be used to stratify patients or predict prognosis, its use as a therapeutic target deserves attention. In fact, a direct inhibition of this transporter may lead to a metabolic shift, generating more aggressive metabolic states in cancer patients, as suggested by the poor outcome in UCEC patients with a low SLC52A3 expression. However, the high SLC52A3 expression in cancer patients could be exploited to deliver riboflavin-conjugated drugs selectively into tumor cells, opening new perspective in treatment of these malignancies.

Our analysis regarding genetic alterations associated with the SLC52A3 gene in gynecological cancers showed that the frequency of SLC52A3 alterations is generally very low (0–4.28%), with the highest alteration frequency observed in ovarian cancer. However, these alterations did not significantly affect overall survival, progression-free survival, or disease-free survival in any of the gynecological cancers studied, demonstrating that SLC52A3 gene alterations are not clinically relevant in gynecological cancers.

Immune infiltration describes the presence and arrangement of immune cells (e.g., lymphocytes) within tumor tissues and is strongly linked to the clinical outcomes in several types [23]. It is generally known that a higher degree of immune cell infiltration correlates with improved prognosis due to the participation of immune cells in aiding the reduction in tumor growth and progression. Our immune infiltration analysis showed a significant association between SLC52A3 expression and the infiltration of immune cells such as cancer-associated fibroblasts (CAFs) and endothelial cells in ovarian and cervical cancers, suggesting that SLC52A3 may influence the immune landscape within the tumor microenvironment (TME) in these malignancies, opening avenues for possible immunotherapy applications. CAFs are key components of the tumor stroma and may promote cancer progression by enhancing extracellular matrix remodeling, the secretion of pro-invasive cytokines and growth factors, immune suppression, angiogenesis, and metastasis [24,25,26]. Therefore, the association between SLC52A3 expression and CAF signatures in ovarian cancer may suggest a potential relationship between SLC52A3-associated transcriptional programs and stromal remodeling. Conversely, the negative association with endothelial cell signatures may reflect tumor-type-specific differences in vascular abundance, stromal composition, tumor purity, or angiogenic activity.

The potential relationship between SLC52A3 expression and the tumor microenvironment is particularly relevant in light of recent evidence linking inflammatory and immune-related biomarkers to prognosis and therapeutic personalization in gynecological malignancies [27]. In fact, in locally advanced cervical cancer treated with definitive chemoradiotherapy, several dynamic changes in serum inflammatory markers, such as the neutrophil-to-lymphocyte ratio (NLR), platelet-to-lymphocyte ratio (PLR), monocyte-to-lymphocyte ratio (MLR), systemic inflammation response index (SIRI), and systemic immune-inflammation index (SII), were significantly associated with overall survival, suggesting that systemic inflammatory status may reflect clinically relevant host–tumor immune interactions. However, the relationship between peripheral inflammatory markers and the actual tumor microenvironment requires further ad hoc studies before using these markers for personalized treatment decisions. Moreover, the efficacy of cancer immunotherapy depends on complex interactions among immune surveillance, immune evasion, tumor heterogeneity, signaling pathways, and the tumor microenvironment [28].

However, our immune infiltration results should be interpreted as preliminary evidence. In fact, these associations require functional validation due to the discrepancies among algorithms that may occur because of differences in cell-type marker selection, statistical modeling, normalization procedures, tumor purity correction, and the ability to distinguish closely related or overlapping stromal cell populations.

The enrichment of SLC52A3-related genes in biological processes such as cell junction organization/assembly and protein localization to the plasma membrane, together with KEGG pathways including endocytosis, tight junctions, and adherens junctions, may provide a biologically plausible link between SLC52A3-associated transcriptional programs and gynecological cancer progression. Cell junction remodeling is a key event during tumor progression, since the disruption of adherens and tight junctions can weaken epithelial cell–cell adhesion, alter epithelial polarity, and promote epithelial–mesenchymal transition, thereby increasing tumor cell motility, invasion, and metastatic dissemination [29]. Endocytosis may also contribute to tumor progression by regulating the internalization, recycling, and degradation of membrane receptor proteins involved in oncogenic signaling and cell migration [30,31]. Therefore, the association of SLC52A3-related genes with these pathways suggests that SLC52A3 expression may be linked to epithelial architecture remodeling and invasive behavior in gynecological cancers.

Our analysis also found that SLC52A3 expression was positively correlated with several genes implicated in tumor progression, proliferation, adhesion, immune modulation, and metastasis, including NECTIN4, PROM2, TACSTD2, PKP3, SEMA4B, and CD46 [32,33,34,35,36,37]. Although a direct functional interaction among SLC52A3, NECTIN4, TACSTD2, PROM2, PKP3, and CD46 has not been demonstrated, the positive correlations observed in our analysis may reflect an epithelial membrane-associated transcriptional program relevant to gynecological cancer progression. In support of this interpretation, NECTIN4 has been implicated in ovarian cancer cell adhesion, cancer cell migration, proliferation, and EMT-related phenotypes [38], while TACSTD2/TROP2 overexpression has been associated with aggressive clinicopathological features and poor prognosis in ovarian [39] and endometrial [40] cancers. PKP3, a desmosomal plaque protein, has been linked to ovarian cancer invasion [41], while PROM2 has been associated with paclitaxel resistance in endometrial cancer [42]. Moreover, CD46 may contribute to tumor immune evasion and has been associated with adverse prognostic features in cervical [43] and ovarian [44] cancers. Therefore, these genes should be interpreted as components of a putative SLC52A3-associated co-expression signature rather than as direct downstream effectors of SLC52A3. Further validation in independent transcriptomic cohorts and functional knockdown or overexpression experiments will be required to determine whether SLC52A3 and these cancer-associated genes participate in common regulatory pathways driving invasion, metastasis, or epithelial–mesenchymal transition in gynecological cancers.

### Translational Perspectives and Potential Clinical Application

Several validation steps are required for a translational perspective of SLC52A3 as a biomarker. First, the association between SLC52A3 expression and prognosis should be confirmed in independent cohorts of patients with gynecological cancers, particularly in UCEC patients, where we observe the strongest survival association. Second, clinically applicable cut-off values should be established together with clinicopathological parameters such as tumor stage, histological subtype, grade, molecular subtype, and treatment regimen. Third, the analytical reproducibility of SLC52A3 detection should be evaluated using standardized technologies routinely used in pathology workflows.

This stepwise validation strategy is consistent with recent translational biomarker studies showing that candidate biomarkers require structural or molecular confirmation, standardized quantitative assays, reference range definition, and evaluation of their relationship with clinically meaningful outcomes before clinical implementation [45,46,47,48,49].

In this context, SLC52A3 could initially be assessed in tumor tissue using immunohistochemistry and RNA in situ hybridization since these analyses preserve tissue architecture and may help determine whether SLC52A3 expression is mainly tumor cell intrinsic or influenced by stromal, endothelial, or immune cell components of the tumor microenvironment.

Beyond conventional laboratory analysis, biosensor-based platforms may provide future opportunities for rapid, sensitive, and potential point-of-care biomarker detection. In fact, these biosensors can detect nucleic acids, proteins, metabolites, extracellular vesicles, and circulating tumor-associated biomarkers with high sensitivity and specificity [50]. Therefore, once SLC52A3 is experimentally validated at the tissue and protein levels, biosensor-based strategies could theoretically be explored for the detection of SLC52A3 mRNA, SLC52A3 protein, or SLC52A3-associated molecular signatures in tumor tissue, liquid biopsy samples, extracellular vesicles, uterine aspirates, or cervicovaginal fluids. However, it deserves to be pointed out that these applications remain speculative at present and would require rigorous analytical validation and comparison with gold-standard assays.

Therefore, the most realistic clinical application for SLC52A3 would not be its immediate use as a stand-alone biomarker but rather its incorporation into a multi-marker prognostic model integrating transcriptomic, histopathological, immune infiltration, metabolic, and clinical variables. Using SLC52A3 in such a multi-marker prognostic model could potentially help to identify patients at higher risk of recurrence or poor outcome, guiding surveillance intensity. Moreover, such a model would support future studies evaluating potential treatment personalization. However, prospective clinical trials and functional studies are required before SLC52A3 can be considered for decision-making in routine gynecologic oncology.

## 5. Limitations

The data reported in our analysis primarily relied on publicly available datasets such as TCGA_GTEx, which may contain limitations related to sample diversity. Thus, the major limitation of this study is that the findings are derived from public transcriptomic and multi-omics databases and are therefore primarily descriptive and hypothesis-generating. Although the observed associations between SLC52A3 expression, survival outcomes, immune infiltration estimates, and enrichment pathways suggested potential biological relevance, they do not establish causality. Functional studies are required to determine whether and how SLC52A3 regulates tumor progression, metabolic adaptation, mitochondrial function, EMT, invasion, or tumor microenvironment remodeling.

Future studies should include SLC52A3 knockdown and overexpression in gynecological cancer cell lines, followed by assays of proliferation, apoptosis, colony formation, migration, invasion, EMT marker expression, riboflavin uptake, mitochondrial respiration, oxidative stress, and metabolomic profiling of riboflavin/FMN/FAD-dependent pathways. Furthermore, validation in independent patient cohorts and analysis using single-cell or spatial transcriptomic datasets will be important to determine whether SLC52A3 expression is tumor-cell intrinsic or influenced by stromal, immune, or endothelial cell populations. Ultimately, these studies may help to determine the potential role of SLC52A3 as a reliable biomarker or a potential therapeutic target in gynecological cancers.

## 6. Conclusions

Our study suggested an important role of SLC52A3 in gynecological cancers since it may influence tumorigenesis through its involvement in key signaling pathways and in the immune landscape within the TME. According to our results, SLC52A3 may have biological and prognostic relevance in gynecological cancers, particularly in UCEC. However, its clinical implementation requires further validation in independent cohorts, standardization of detection methods, definition of clinically meaningful cut-off values, and integration with established clinicopathological and molecular parameters. Thus, the present study should therefore be interpreted as a bioinformatic, hypothesis-generating analysis that provides a rationale for future mechanistic studies since the functional role and therapeutic relevance of SLC52A3 remain to be experimentally established.

## Data Availability

The datasets generated during and/or analyzed during the current study are available from the corresponding author on reasonable request.

## Abbreviations

The following abbreviations are used in this manuscript:

ATP	Adenosine Triphosphate
BioGRID	Biological General Repository for Interaction Datasets
CAFs	Cancer-Associated Fibroblasts
C20orf54	Chromosome 20 Open Reading Frame 54
CESC	Cervical Squamous Cell Carcinoma
CNA	Copy Number Alteration
CONSENSUS_TME	Consensus Tumor Microenvironment estimation
cBioPortal	cBio Cancer Genomics Portal
CYC1	Cytochrome c-1
DFS	Disease-Free Survival
EPIC	Estimation of Proportions of Immune and Cancer Cells
FAD	Flavin Adenine Dinucleotide
FMN	Flavin Mononucleotide
GEPIA2	Gene Expression Profiling Interactive Analysis (version 2)
GEPIA3	Gene Expression Profiling Interactive Analysis (version 3)
GO	Gene Ontology
GTEx	Genotype-Tissue Expression
HPA	Human Protein Atlas
KEGG	Kyoto Encyclopedia of Genes and Genomes
KM	Kaplan–Meier
MCPCOUNTER	Microenvironment Cell Populations Counter
mRNA	Messenger RNA
OS	Overall Survival
OV	Ovarian Cancer
PCC	Pearson Correlation Coefficient
PFS	Progression-Free Survival
PFI	Progression-Free Interval
SLC52A3	Solute Carrier Family 52 Member 3
SRplot	Scientific Research Plotting platform
STRING	Search Tool for the Retrieval of Interacting Genes/Proteins
TCGA	The Cancer Genome Atlas
TIDE	Tumor Immune Dysfunction and Exclusion
TIMER2	Tumor Immune Estimation Resource (version 2)
TIMER3	Tumor Immune Estimation Resource (version 3)
TME	Tumor Microenvironment
TPM	Transcripts Per Million
UCEC	Uterine Corpus Endometrial Carcinoma
UCS	Uterine Carcinosarcoma
UQCR10	Ubiquinol-Cytochrome C Reductase Complex III Subunit X
UQCRH	UQCR Hinge Protein
XCELL	Cell Type Enrichment Analysis
